# Entropy Rate Superpixel Classification for Automatic Red Lesion Detection in Fundus Images

**DOI:** 10.3390/e21040417

**Published:** 2019-04-19

**Authors:** Roberto Romero-Oraá, Jorge Jiménez-García, María García, María I. López-Gálvez, Javier Oraá-Pérez, Roberto Hornero

**Affiliations:** 1 Biomedical Engineering Group, E.T.S.I. de Telecomunicación, University of Valladolid, 47011 Valladolid, Spain; 2 Department of Ophthalmology, Hospital Clínico Universitario de Valladolid, 47003 Valladolid, Spain; 3 Instituto de Oftalmobiología Aplicada (IOBA), University of Valladolid, 47011 Valladolid, Spain; 4 Instituto de Investigación en Matemáticas (IMUVA), University of Valladolid, 47011 Valladolid, Spain; 5 Instituto de Neurociencias de Castilla y León (INCYL), University of Salamanca, 37007 Salamanca, Spain

**Keywords:** diabetic retinopathy, retinal imaging, red lesion, entropy rate superpixel segmentation, multilayer perceptron

## Abstract

Diabetic retinopathy (DR) is the main cause of blindness in the working-age population in developed countries. Digital color fundus images can be analyzed to detect lesions for large-scale screening. Thereby, automated systems can be helpful in the diagnosis of this disease. The aim of this study was to develop a method to automatically detect red lesions (RLs) in retinal images, including hemorrhages and microaneurysms. These signs are the earliest indicators of DR. Firstly, we performed a novel preprocessing stage to normalize the inter-image and intra-image appearance and enhance the retinal structures. Secondly, the Entropy Rate Superpixel method was used to segment the potential RL candidates. Then, we reduced superpixel candidates by combining inaccurately fragmented regions within structures. Finally, we classified the superpixels using a multilayer perceptron neural network. The used database contained 564 fundus images. The DB was randomly divided into a training set and a test set. Results on the test set were measured using two different criteria. With a pixel-based criterion, we obtained a sensitivity of 81.43% and a positive predictive value of 86.59%. Using an image-based criterion, we reached 84.04% sensitivity, 85.00% specificity and 84.45% accuracy. The algorithm was also evaluated on the DiaretDB1 database. The proposed method could help specialists in the detection of RLs in diabetic patients.

## 1. Introduction

Diabetic retinopathy (DR) is a microvascular complication of diabetes leading to vision loss. It is the most common cause of blindness in the working-age population in developed countries [1,2]. Since treatment is only effective in the early asymptomatic stages, regular follow-up has proven essential to prevent sight damage [3,4]. As digital color fundus images are low-cost and patient friendly, they are commonly used for large-scale screening [4]. The analysis of these photographs allows the detection of the lesions associated with DR, including microaneurysms (MAs), hemorrhages (HEs), exudates (EXs), and cotton wool spots (CWs) [5].

With the limited number of specialists and the increasing prevalence of diabetes, automation in the screening process could be beneficial, since it may reduce the workload of ophthalmologists and the health costs [2,6].

Red lesions (RLs) are among the first signs of DR. Consequently, RL detection is an important step in the automated screening of DR [3]. RLs include MAs and HEs. MAs are balloon-shaped alterations of the vessel walls caused by the abnormal permeability of the vasculature due to hyperglycemia [7]. HEs are bigger red spots with irregular margin and/or uneven density produced by leakages of weak capillaries [7].

Numerous studies can be found in the literature regarding RL detection. There are studies focused on exclusively detecting MAs and others on exclusively detecting HEs. We can also find that MAs and HEs are frequently detected together since they are difficult to differentiate for clinicians [2]. Early studies used fluoroscein angiograms [8,9,10,11] and red-free photographs [12]. Later, the use of color fundus images was extended [2,7]. Different methods have been proposed in the literature to detect RLs in color fundus images. Those focused exclusively on detecting MAs have shown the effectiveness of mathematical morphology, region growing and wavelets among other techniques. Thus, they can be divided into four groups [7]:

- *Mathematical morphology-based methods*. The earliest study in MA detection used a top-hat transformation over fluoroscein angiograms [8]. Walter et al. [13] detected MA candidates applying a top-hat transformation together with global thresholding. Then, they contributed by adding a stage of candidate classification extracting fifteen features and applying *k*-nearest neighbors (*k*-NN), Gaussian and kernel density estimation-based classifiers over the candidates.
- *Region growing-based methods*. Fleming et al. [14] applied contrast normalization, watershed gradient and region growing in addition to a *k*-NN classifier using nine features from candidate regions. Other studies introduced the use of adaptive thresholding and the moat operator [15].
- *Wavelet-based*. Quellec et al. [16] found it useful to apply a wavelet transform to locally match a MA template. Wavelets in fundus image compression have also been studied, showing that the Industry standard Joint Photographic Experts Group (JPEG) compression is suitable to detect MAs [17].
- *Hybrid approaches*. Spencer et al. [9] used mathematical morphology and a matched filter over fluorescein angiograms. This method combined a Gaussian filter, a top-hat transformation and region growing applied after a novel matched-filtering. This work was extended in another approach by applying an alternative region growing and classification algorithms [10]. In the same way, Frame et al. [11] based their work on [9] correcting the illumination variations and applying Linear Discriminant Analysis, neural networks (NN) and rule-based classifiers. Niemeijer et al. [3] introduced a method based on pixel classification using *k*-NN. They removed the vasculature and extracted a set of features extended from [9] and [11]. Other approaches contributed by using a NN to detect MAs [18]. Locally adaptive contrast enhancement was applied and the optic disc and vessels were segmented. Then, features related to size, shape, hue and intensity were extracted for the classification. Lazar and Hajdu [19] employed a directional cross-section profile centered on the local maximum of the image pixels. Size, shape and height features were extracted after peak detection. Finally, a Naive Bayes classifier was used.

Other approaches focused on detecting HEs alone. They can be divided into two categories [7]:

- *Mathematical morphology*. Hatanaka et al. [20] presented a method based on brightness correction over the Hue–Saturation–Value (HSV) space and thresholding. Another algorithm used novel multiscale and morphological techniques [21]. Median filtering and histogram equalization were applied during preprocessing. Furthermore, morphological operations were performed. Fleming et al. [14] detected large abnormal regions by iteratively applying various multiscale structuring elements. However, small dark lesions were not easily detected.
- *Pixel classification*. Zhang and Chutatape detected HEs using Support Vector Machine [22]. The main contribution was the extraction of features using Principal Component Analysis.

Although extensive previous research has been devoted to the detection of MAs and HEs separately, these lesions can also be detected together. This is because their similar color makes it difficult for automatic systems and clinicians to differentiate them [2]. In addition, the joint detection of MAs and HEs is sufficient to determine the presence of DR [2]. The works where MAs and HEs are detected together propose varied techniques. In this context, Seoud et al. [23] proposed a technique based on dynamic shape features representing the evolution of the shape during image flooding. Some recent studies based on deep learning have also been published. Some of them combined features extracted by means of convolutional neural network (CNN) together with hand crafted features and a random forest classifier [24].

Almost all previous methods consider pixels as the basic unit in the image. However, another novel perspective based on superpixel segmentation can be found in the literature [25]. A superpixel is a perceptually uniform region in the image. While pixels provide a discrete representation of the image, superpixels represent natural entities [25,26]. In contrast to pixel representation, superpixel approach reduces the number of basic units, which improves the computational efficiency. It also provides the spatial support for computing region-based features [27]. Additionally, superpixel segmentation is more consistent with human visual cognition and contains less redundancy [25,26].

In this sense, Zhou et al. [25] calculated multichannel multifeatures over a superpixel segmentation based on the Simple Linear Iterative Clustering (SLIC) technique. It should be noted that, in this study, blood vessels were removed separately using multiscale morphology. However, it is very difficult to accurately segment the blood vessels and some parts tend to be disconnected. As a consequence, the need to remove the blood vessels before superpixel classification is applied leads to the incorrect detection of blood vessel segments as RLs. In our previous work [28], the SLIC algorithm was also applied to extract the superpixels from an image. The SLIC method has been widely used in image processing but has important limitations in terms of accuracy and boundary adherence [29]. Frequently, it produces some under-segmented superpixels [29]. We propose a novel RL detection method based on superpixel classification which avoids the need for independent blood vessel removal, unlike most RL detection methods. Consequently, we avoid the possible errors derived from this phase. Our approach is based on several stages. First, a novel preprocessing technique is proposed in order to achieve intra-image and inter-image normalization. It includes several operations for retinal image enhancement: bright border artifact removal, retinal fundus extension outside the field-of-view (FOV), intra-image and inter-image illumination and color equalization, denoising and contrast enhancement. To the best of our knowledge, this is the first time that the proposed operations have been combined in a pipeline. This step is paramount to obtain an accurate superpixel extraction in the subsequent stages. It should also be noted that the proposed preprocessing algorithm could be useful for different retinal image analysis problems, beyond RL detection. Then, candidate regions were segmented, where the Entropy Rate Superpixel (ERS) Segmentation method was introduced. To the best of our knowledge, it has not been previously used for retinal image analysis. ERS is an efficient greedy algorithm which follows a clustering formulation based on an entropy measure [27]. Under condition of the same initial number of superpixels, the performance comparisons show that the ERS method overcomes the SLIC method in the three standard superpixel segmentation metrics [29]: Boundary Recall [30], Under-segmentation Error [26] and Achievable Segmentation Accuracy [30]. Boundary Recall measures the level of coincidence between the superpixel segmentation boundary and the ground truth boundary. Under-segmentation Error evaluates the quality of segmentation boundary by punishing the superpixel for overlapping multiple objects. Achievable Segmentation Accuracy is defined as the upper bound of the object segmentation accuracy that can be achieved [29]. Based on our experiments, we have found that the ERS method is highly configurable for the generation of superpixels. Thus, the appropriate selection of the parameters in this method allowed us to perfectly adjust the superpixels to the shapes of the lesions. This resulted in a very precise segmentation of RL, closer to the expert annotations than the one obtained with the SLIC method. Next, the segmented superpixels were combined to reduce inaccurate fragmentation within structures. In the next stage, multiple features on such regions were extracted and selected using the Fast Correlation Based Filter (FCBF) technique. Finally, a MultiLayer Perceptron (MLP) was used to distinguish the true RLs from the rest of the candidates.

The paper is organized as follows. In Section 2, the proposed method is detailed. The experimental results of the study obtained according to two different criteria are presented in Section 3 together with the description of the retinal image database. Finally, the discussion and conclusions of the study are drawn in Section 4.

## 2. Proposed Method

**Figure 1:**
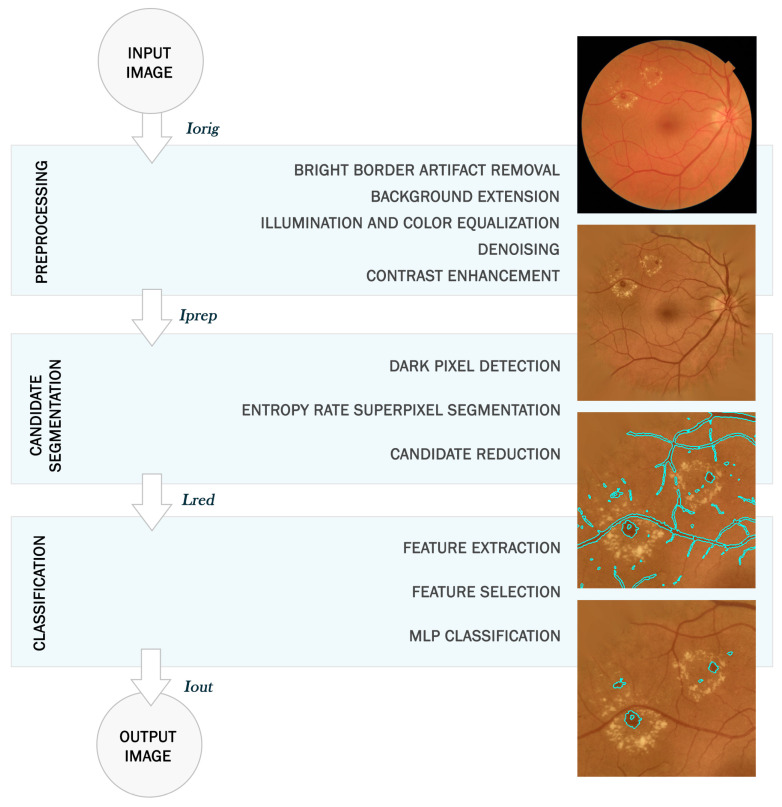
Block diagram of the proposed approach.

The proposed method comprises three sequential stages in order to obtain the final RL segmentation, as shown in Figure 1. Each of these stages is explained in the following subsections.

### 2.1. Preprocessing

The appearance of the fundus images is strongly correlated to the intrinsic features of the patient, such as his skin or iris color, among others [6,31]. In addition, local illumination and contrast are often non-uniform within the retinas. Therefore, we can find a wide variability of images in terms of color, luminosity, contrast and quality [6].

When processing fundus images, it is important that the FOV is separated from the surrounding black border. This way, only the pixels of the image belonging to the retinal area would be processed. In this study, the FOV was automatically determined from the original image, $I_{orig}$, as part of the proposed method, prior to preprocessing. The diameter, *D*, was estimated analyzing the intensity profile along one image diagonal in the red component [32]. Then, FOV edges were found using a Canny edge detector and the FOV center was estimated with a circular Hough transform [32].

**Figure 2:**
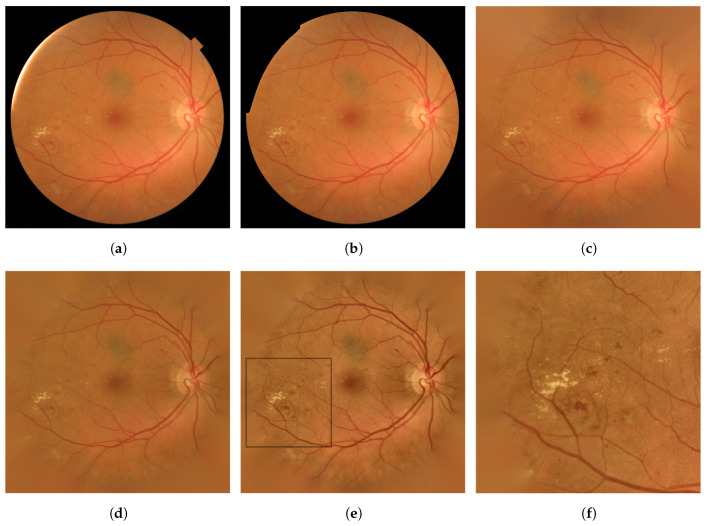
Preprocessing stage: (**a**) original image, $I_{orig}$; (**b**) image after bright border artifact removal, $I_{rem}$; (**c**) effect of background extension, $I_{ext}$; (**d**) result after illumination and color equalization, $I_{eq}$; (**e**) final preprocessed image with contrast enhancement, $I_{prep}$; (**f**) zoom of the final preprocessed image.

After FOV extraction, we performed a preprocessing stage aimed at achieving intra-image and inter-image normalization. Additionally, we tried to enhance retinal landmarks, with a special attention on improving the visualization of RLs. For this task, we performed five sequential operations on $I_{orig}$, shown in Figure 2a:

- *Bright border artifact removal.* Some images present excessively bright regions along the FOV border. This is generally due to inadequate illumination during image acquisition. While they do not prevent image visualization, these bright regions may be problematic in the remaining stages of the proposed method due to their color and border features [33]. Bright border artifacts were detected using the blue channel, $B_{orig}$, in the Red-Green-Blue (RGB) color space [33]. First, a mask of bright pixels was calculated as $B_{mask}=B_{orig}-B_{mean}$, where $B_{mean}$ is a mean-filtered version of $B_{orig}$ [33]. Second, a morphological reconstruction was applied using the FOV contour as the marker and $B_{mask}$ as the mask [33]. Then, the binary mask of the bright border artifacts was obtained by a threshold followed by a morphological opening [33]. Figure 2b shows the resulting image after the artifact removal, $I_{rem}$.
- *Background extension.* The strong contrast between the retinal fundus and the black region outside the aperture may yield edge effects in later operations [34]. For each RGB channel of $I_{rem}$, retinal fundus was extended outside of the FOV with an iterative algorithm. Pixels outside the aperture were replaced with the mean value of the neighboring pixels inside the FOV [34]. The result of this operation, $I_{ext}$, can be seen in Figure 2c.
- *Illumination and color equalization.* Retinal images have often non-uniform illumination and different color ranges. Equalizing illumination as well as overall color is necessary to obtain standardized images. First of all, $I_{ext}$ was converted to Hue–Saturation–Intensity (HSI) color space. Local variations of intensity within the image were equalized over the intensity channel, $I_{ext_{I}}$, by applying [35]:
  (1) $$I_{eq_{I}}=I_{ext_{I}}+\mu -I_{ext_{I}}*h_{m1}.$$
  In this equation, $h_{m1}$ is a large mean filter, similar in size to the optic disc [23]. The parameter μ is the average pixel intensity in $I_{ext_{I}}$ inside the FOV of the images of the training set. This allowed us to normalize the overall illumination among images. To normalize hue and saturation channels, $I_{ext_{H}}$/$I_{ext_{S}}$, we applied:
  (2) $$I_{eq_{color}}=I_{in}+\mu_{1}-\mu_{2},$$
  where $\mu_{1}$ is the average pixel intensity in $I_{in}$ inside the FOV of the images of the training set and $\mu_{2}$ is the average pixel intensity inside the FOV of the input image, $I_{ext_{H}}/I_{ext_{S}}$. Taking into account that $I_{ext_{H}}$ represents an angle, the following equation needs to be applied to calculate the average hue:
  (3) $$\mu_{hue}=atan2\left[\sum_{i=1\ldots N}\sin I_{ext_{H}}\left(i\right),\sum_{i=1\ldots N}\cos I_{ext_{H}}\left(i\right)\right],$$
  where *N* is the total number of pixels in the image. Finally, converting the HSI image back to the RGB space, we obtained the image $I_{eq}$ shown in Figure 2d.
- *Denoising.* This step allowed us to reduce the noise associated with image capture and compression. The noise was eliminated by applying an additional mean filter over $I_{eq}$ to obtain $I_{den}$ [23]. This mean filter had to be small in size in order to keep the shape of the smallest RLs. In this study, we empirically chose a filter size of 3 pixels [23].
- *Contrast enhancement.* Some retinal images present poor contrast. Therefore, the RLs can not be clearly seen. The contrast limited adaptive histogram equalization (CLAHE) method was applied to enhance local contrast. This is a histogram processing technique that operates on small regions. This operation highlighted the edges of the RLs to be later segmented [36]. After the contrast enhancement, we obtained the final preprocessed image $I_{prep}$. The result can be seen in Figure 2e. Figure 2f shows an enlarged region of the image, where lesions are present.

**Figure 3:**
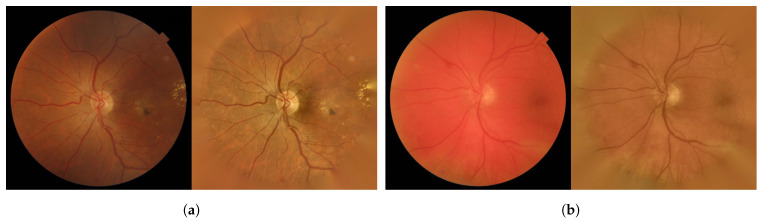
Preprocessing for fundus images with different illumination, contrast and color. (**a**) Example 1; (**b**) example 2.

The different operations in the preprocessing stage allowed us to highlight RLs and avoid border effects in subsequent stages. It also allowed us to perform inter-image normalization. An example can be seen in Figure 3, where two original images from our database and their corresponding preprocessed images are shown.

For a better understanding of the complete preprocessing method, the following pseudocode in Algorithm 1 is provided:

**Algorithm 1:** Preprocessing1 $I_{rem}$ = brightBorderArtifactRemoval($I_{orig}$); 2 $I_{ext}$ = backgroundExtension($I_{rem}$); 3 $I_{ext_{HSI}}$ = $I_{ext}$ → HSI color space; 4 $I_{eq_{I}}$ = $I_{ext_{I}}$ + μ − $I_{ext_{I}}$ ∗ $h_{m1}$; 5 $I_{eq_{H}}$ = $I_{ext_{H}}$ + $\mu_{1_{H}}$ − $\mu_{2_{H}}$; 6 $I_{eq_{S}}$ = $I_{ext_{S}}$ + $\mu_{1_{S}}$ − $\mu_{2_{S}}$; 7 $I_{eq}$ = ($I_{eq_{I}}$, $I_{eq_{H}}$, $I_{eq_{S}}$) → RGB color space; 8 $I_{den}$ = meanFilter($I_{eq}$); 9 $I_{prep}$ = CLAHE($I_{den}$);

### 2.2. Candidate Segmentation

The aim of this stage was to separate the candidate RL regions. Since RLs appear as dark regions in contrast with the background, we considered every dark region to be a candidate [3]. Once the preprocessed image was obtained, we carried out the segmentation of dark regions in three steps. First, we detected the dark pixels in the image. Second, we grouped the pixels in the image in superpixels. Finally, we reduced the number of candidate regions to exclusively keep the dark ones.

#### 2.2.1. Dark Pixel Detection

**Figure 4:**
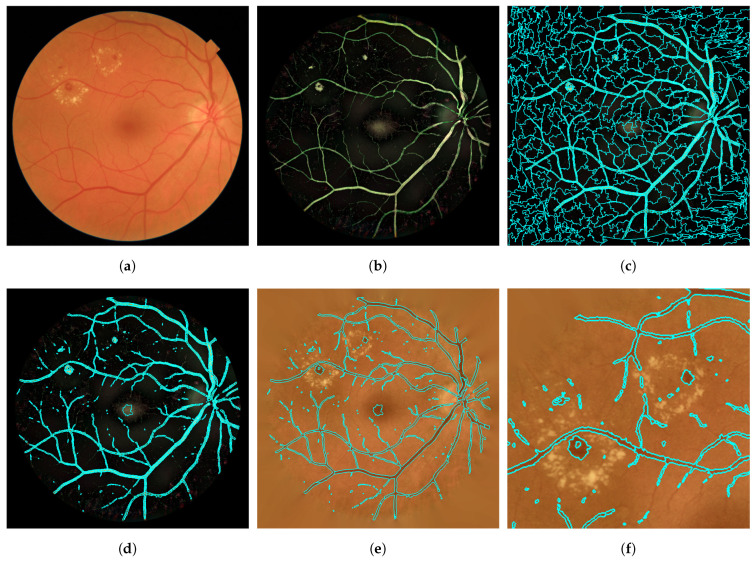
Candidate segmentation. (**a**) Original image with some RLs; (**b**) dark pixels detection computed using the multiscale algorithm; (**c**) segmented superpixels using the Entropy Rate Superpixel method; (**d**) reduced candidates on $I_{dark}$; (**e**) combined, reduced candidates shown over $I_{prep}$; (**f**) zoom of combined, reduced candidates shown over $I_{prep}$.

We detected the dark pixels using a multiscale algorithm on $I_{prep}$ to obtain the image $I_{dark}$. For this task, a two-step process was carried out [28]:

1. An initial estimate of the bright pixels of $I_{prep}$ was calculated, obtaining the image $I_{bri}$. This image was used in the next step to prevent the edges of the bright structures from being considered as dark regions. The Alternating Sequential Filtering (ASF) method was applied on $I_{prep}$ to roughly remove all of the dark pixels from the image (mainly blood vessels and RLs) [33]. As a result, the image $I_{asf}$ was obtained. Subsequently, a multiscale operation was applied over the three color channels of $I_{asf}$ to obtain $I_{bri}$ [28]:
  (4) $$I_{bri}=\max_{s}\left(\alpha_{s}\left(I_{asf}-I_{bg,s}\right)\right).$$
  The parameter *s* represents a scale that depends on *D* (FOV diameter). In this study, $s=\left\{\frac{D}{48},\frac{D}{24},\frac{D}{12},\frac{D}{6},\frac{D}{3}\right\}$. The parameter $I_{bg,s}$ is the background of $I_{asf}$, estimated with a mean filter of size *s*. Finally, the parameter $\alpha_{s}$ was empirically calculated as [28]:
  (5) $$\alpha_{s}=1-\left(3.84\frac{s}{D}\right).$$
2. Dark pixels in the image were detected to obtain $I_{dark}$ [28]. This image represents the level of darkness of the pixels with respect to the background of the retina. Similar to Equation (4), we calculated $I_{dark}$ applying [28]:
  (6) $$I_{dark}=\max_{s}\left(\alpha_{s}\left(\left(I_{prep}-I_{bri}\right)-I_{bg,s}\right)\right).$$
  The term $I_{prep}-I_{bri}$ prevents false detections of dark pixels at the edges of the bright structures. We considered all three color channels avoiding losing information. This highlighted the color difference of the dark pixels in contrast to the background, as can be seen in Figure 4b.

#### 2.2.2. Entropy Rate Superpixel Segmentation

The next step was to group the pixels of $I_{dark}$ into superpixels that separated the different elements of the image. A superpixel is a perceptually uniform region in the image. It groups pixels with similar color and texture and adapts to the image borders [25]. Since it represents natural entities in the image, the superpixel is an appropriate region from which to extract features [25]. In addition, it reduces the complexity of subsequent image processing tasks and improves the computational efficiency [26,27].

We used the ERS method, which is regarded as a clustering problem solved using graph partitioning [27]. First, an undirected graph was constructed mapping its vertices ($V=\{v_{1},v_{2},\ldots \}$) to the pixels of $I_{dark}$. The weights (*w*) of the edges ($E=\{e_{1},e_{2},\ldots \}$) of the graph were the pairwise similarities between vertices. They were calculated using a Gaussian kernel as [27]:

(7) $$w_{i,j}=e^{-\frac{d{\left(v_{i},v_{j}\right)}^{2}}{2\sigma }},$$

where σ is the user-defined Gaussian kernel bandwidth and $d(v_{i},v_{j})$ is the intensity difference between pixels $v_{i}$ and $v_{j}$ multiplied by the spatial distance between them. Since the graph was undirected, the edge weights were symmetrical ($w_{i,j}=w_{j,i}$) [27]. In addition, every vertex of the graph had a self loop. Then, a subset of edges A⊆E was selected such that the resulting graph contained exactly *K* connected disjoint subgraphs $\left\{S_{1},S_{2},\ldots ,S_{K}\right\}$. These subgraphs must also satisfy that $S_{i}\cap S_{j}=⌀$ for i≠j and $\cup_{i}S_{i}=V$ [27]. For the non-selected edges, the edge weight of the self loop of the associated vertices was increased so that the total incident weight for each vertex remained constant [27].

Thus, the graph partitioning problem results in the selection of a subset of edges A that represent the superpixels in the image. For this task, a sequential selection of edges is carried out following the approach of a particle random walk through the graph [37]. In the first step, the particle is positioned at a random node of the graph. Then, the particle randomly moves from node to node forming a sequence of vertices [37]. At every step, the next vertex is chosen from among the nodes connected to the last vertex of the sequence with a probability proportional to the weight of the edge [37]. In this way, we searched for the graph topology that maximized the following objective function [37]:

(8) $$\max_{A}H\left(A\right)+\gamma B\left(A\right),$$

subject to A⊆E and $N_{A}\geq K$, being $N_{A}$ the number of connected vertices in the graph. The parameter H(A) is the entropy rate, defined as [27]:

(9) $$H\left(A\right)=-\sum_{i}\mu_{i}\sum_{j}p_{i,j}\left(A\right)log\left(p_{i,j}\left(A\right)\right),$$

where $\mu_{i}$ is [27]:

(10) $$\mu_{i}=\frac{w_{i}}{\sum_{i=1}^{\left|V\right|}w_{i}},$$

and $p_{i,j}$ is the transition probability [27]:

(11) $$p_{i,j}\left(A\right)=\left\{\begin{array}{cc} \frac{w_{i,j}}{w_{i}}, & \;\mathrm{if}\;i\neq j\;\mathrm{and}\;e_{i,j}\in A,\\ 0, & \;\mathrm{if}\;i\neq j\;\mathrm{and}\;e_{i,j}\notin A,\\ 1-\frac{\sum_{j:e_{i,j}\in A}w_{i,j}}{w_{i}}, & \;\mathrm{if}\;i=j, \end{array}\right.$$

where $w_{i}$ is the sum of incident weights to the vertex $v_{i}$.

Additionally, the parameter B(A) in (8) is called the balancing function. It is defined as [27]:

(12) $$B\left(A\right)=-\sum_{j}p_{Z_{A}}\left(i\right)log\left(p_{Z_{A}}\left(i\right)\right)-N_{A},$$

where $p_{Z_{A}}$ is a probability distribution defined as [27]:

(13) $$p_{Z_{A}}\left(i\right)=\frac{\left|S_{i}\right|}{\left|V\right|},i=\left\{1,\ldots ,N_{A}\right\}.$$

The remaining parameter in (8) is the weight of the balancing term, γ≥0, given by [27]:

(14) γ=βKλ,

where β is defined as: [27]:

(15) $$\beta =\frac{\max_{e_{i,j}}H\left(e_{i,j}\right)-H\left(⌀\right)}{\max_{e_{i,j}}B\left(e_{i,j}\right)-B\left(⌀\right)}.$$

In this equation, the difference $H\left(e_{i,j}\right)-H\left(⌀\right)$ refers to the entropy rate increase when including a single edge into the graph. Likewise, the difference $B\left(e_{i,j}\right)-B\left(⌀\right)$ refers to the balancing term increase when including a single edge into the graph. Therefore, β is the ratio of the maximal entropy rate increase and the maximal balancing term increase upon including a single edge into the graph.

**Figure 5:**
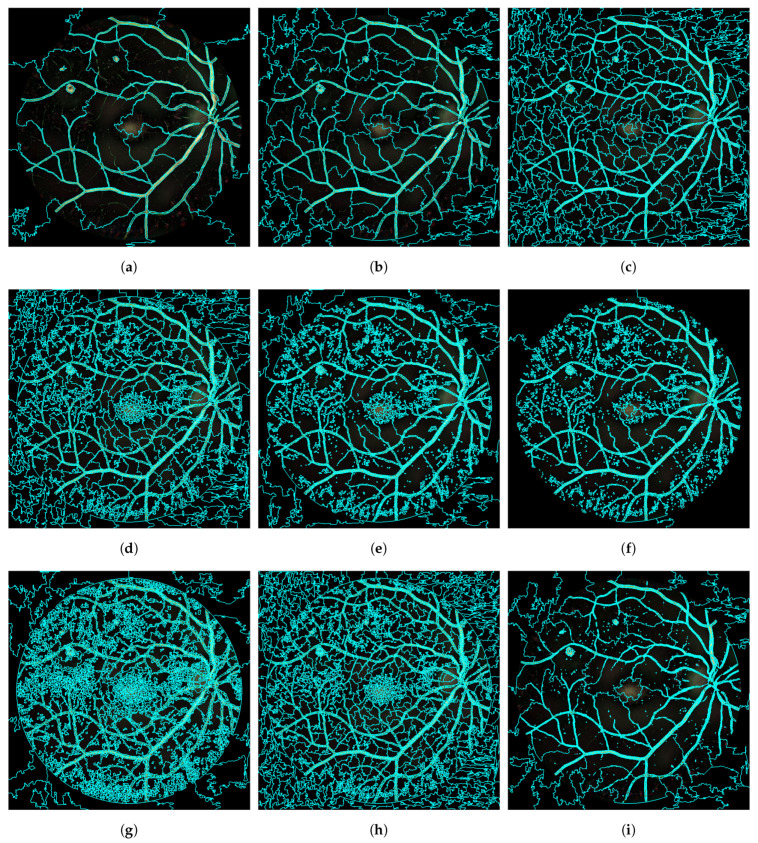
Entropy Rate Superpixel Segmentation with different parameter values. (**a**) K=100, λ=0.1 and σ=5; (**b**) K=500, λ=0.1 and σ=5; (**c**) K=2000, λ=0.1 and σ=5; (**d**) K=2000, λ=0.8 and σ=2; (**e**) K=2000, λ=0.01 and σ=2; (**f**) K=2000, λ=0.001 and σ=2; (**g**) K=2000, λ=0.1 and σ=0.5; (**h**) K=2000, λ=0.1 and σ=2; (**i**) K=2000, λ=0.1 and σ=5.

It should be noted that the parameter H(A) favors the formation of compact and homogeneous clusters. It makes the superpixels adjust to the edges of the structures, resulting in the division of images on perceptual boundaries [27]. However, in most cases, it induces several superpixels to overlap with a single object. To overcome this issue, B(A) is introduced. This parameter encourages the formation of clusters (superpixels) with similar sizes [27]. Another parameter that should be taken into account is *K*, which favors the balancing term [27]. Additionally, β compensates for the magnitude difference H(A) and B(A) [27]. Finally, λ is a user-specified constant and should be empirically determined [27]. Applying ERS to fundus images requires properly adjusting the parameters *K*, λ and σ. *K* should be large enough to isolate every structure, paying special attention to the smallest RLs. Meanwhile, the parameter σ should be selected to ensure that the superpixels adjust robustly to the edges of the objects in the image. λ should compensate the fragmentation effect to avoid over-segmentation within retinal structures. In this study, these parameters were experimentally adjusted using the images in the training set. The result of this stage is an image map, *L*, of *K* labels that identify the superpixels that form the image. Figure 5 gives the superpixel segmentation results with different values for each parameter. As shown in Figure 5a, we properly separated the large HEs with K=100. However, we missed all the MAs. With K=500, as shown in Figure 5b, we did not detect the smallest MAs yet. Therefore, a larger *K* should be selected to detect all the RLs, including MAs. By visual inspection of our results for different values of *K* (see Figure 5a–c), we chose K=2000 as adequate to detect every RL. Regarding the value of λ, we encountered that a large value of this parameter prevented sizeable structures to be over-segmented in tiny regions (see Figure 5d). On the contrary, the smallest MAs were more easily detected when λ was small (see Figure 5e,f). Therefore, we chose λ=0.08 as an optimal commitment value. Regarding σ, we found that, when this parameter was too large, the smallest MAs could not be adequately segmented (see Figure 5i). On the other hand, if σ was too small, the superpixels did not adjust accurately to the edges of the structures. We experimentally found that σ=2 provided a good compromise between both situations (see Figure 5h).

In conclusion, to properly separate the retinal RLs, we considered the following parameter values: K=2000, λ=0.08 and σ=2. The result of this stage on the example image can be seen in Figure 4c.

#### 2.2.3. Candidate Reduction

Most of the superpixels in which the image was divided did not correspond to dark regions. Therefore, those superpixels should not be considered as RL candidates. Reducing the number of candidates simplifies the classification task. To reduce candidates, those superpixels of *L* that were not dark were eliminated, obtaining $L_{red}$. For this task, the average intensity of the pixels of each superpixel was calculated on the green channel of the image $I_{dark}$ and those that did not exceed a threshold equal to 0.3 were removed [28]. This threshold was empirically obtained considering that lower values corresponded to non-RL structures. Since the image $I_{dark}$ represents the level of darkness of the pixels with respect to the background of the retina, it is easy to apply a threshold to eliminate the structures that are clearly not dark. An accurate value for this parameter is not crucial since our objective was to coarsely remove the superpixels in the image whose intensities do not correspond to dark structures. Figure 4d shows $L_{red}$ over the $I_{dark}$ image.

Finally, neighboring superpixels with a similar color were combined in an iterative process. To calculate color similarity among the superpixels, we took the average value of the pixels contained in each superpixel in the image $I_{dark}$. Then, the Euclidean distances between the average values were measured over the CIELAB color space [28]. This color space was designed to approximate human vision, representing the nonlinear response of the eye. Therefore, the relative perceptual difference between any two colors defined in CIELAB can be approximated taking the Euclidean distance between them [38]. In this study, the maximum color distance for two superpixels to be combined was empirically settled to 0.24 during training. Figure 4e shows the definitive RL candidates after this stage.

The following pseudocode summarizes the proposed segmentation technique (Algorithm 2):

**Algorithm 2:** Candidate segmentation technique

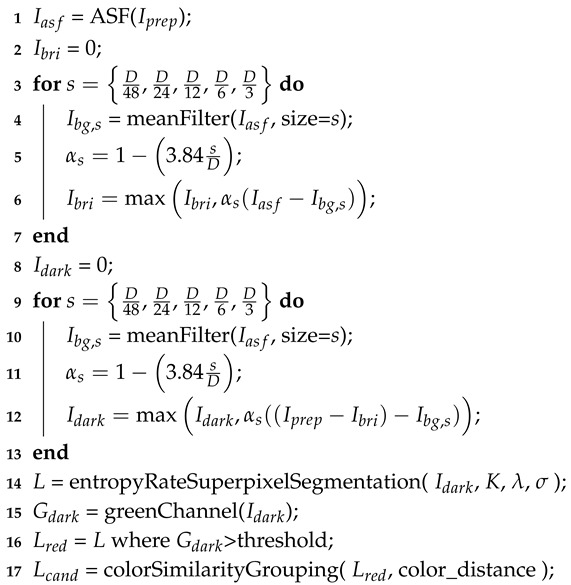



### 2.3. Classification

Once the RL superpixel candidates were obtained, we used an MLP to separate the true RLs from non-RL candidate superpixels. This type of NN has been used in previous studies for the automatic detection of RLs [18,32,39]. The classification stage comprises three procedures:

#### 2.3.1. Feature Extraction

For each superpixel, a set of features was extracted to represent the the visual characteristics of RLs—thus differentiating them from blood vessel segments and other dark regions in the image. Accordingly, we included 39 features specified in Table 1.

#### 2.3.2. Feature Selection

An automated feature selection stage was implemented to avoid redundant information of the extracted features. Reducing the number of features to a set of relevant, low-correlated ones decreases classification errors and simplifies the structure of the classifier [43]. We used the FCBF method, which is a classifier-independent feature selection technique [44]. FCBF computes symmetrical uncertainty (SU) to find the most relevant and non-redundant features for a certain problem. This measure is a normalization of the information gain between two variables, representing the information shared between them. SU is calculated as [44]:

(16) $$SU\left(X_{i},Y\right)=2\left[\frac{H\left(X_{i}\right)-H\left(X_{i}|Y\right)}{H\left(X_{i}\right)+H\left(Y\right)}\right],i=1,2,\ldots ,N,$$

where *N* is the total number of extracted features, H(·) refers to Shannon’s entropy, $X_{i}$ is the *i*th-feature and *Y* is the target (RL or non-RL in our study). FCBF is performed in two consecutive stages [44]:

1. A relevance analysis is carried out by ranking the extracted features according to the values of SU between each of them and the target variable.
2. A redundancy analysis is carried out by computing the SU between each pair of features. Hence, the features that are highly correlated with other relevant features are considered redundant and are removed from the final model.

The result of this stage is a reduced set of the features. The selected features are indicated in the last column of Table 1.

#### 2.3.3. MultiLayer Perceptron Neural Network

Based on the features obtained in Section 2.3.2, supervised learning was applied in order to separate true RLs from non-RLs. We used an MLP, which consists of various fully connected layers of neurons [43,45]. It maps a set of input variables onto a set of output variables using a nonlinear function [43].

A MLP is composed of one input layer, one output layer and, at least, one hidden layer of neurons [43,45]. Optionally, more hidden layers can be added between the input layer and the output layer [43]. However, an MLP with a single hidden layer of neurons is capable of universal approximation [46]. For this reason, we considered a 3-layer MLP. The input layer consisted of as many neurons as features were selected by the FCBF method. The output layer was formed by a single neuron since we tried to resolve a dichotomous problem. The number of hidden units, $n_{hid}$, was experimentally settled during the training stage. The activation function used in the hidden layer was the hyperbolic tangent sigmoid (tanh), which accelerates the learning process of the network [43]. The logistic sigmoid was used as the activation function in the output neuron. Since it is defined in the range (0–1), we could interpret the output of the NN as a posterior probability [43,45].

The training process of the MLP can be viewed as the minimization of an error function. We chose the cross-entropy error function since it simplifies the optimization process when the output layer activation function is the logistic sigmoid [32]. Regarding the learning function used to update the weight and bias values, we applied the scaled conjugate gradient backpropagation method. This algorithm has been shown considerably faster than other supervised learning algorithms [47].

Additionally, a regularization process was employed to penalize large weights during the learning process [43]. This allowed us to avoid overfitting and improve generalization [43]. The regularization parameter, υ, was experimentally determined during training.

## 3. Experimental Results

### 3.1. Retinal Image Databases

We conducted our experiments using two retinal image databases with different characteristics. First, we employed a private dataset. It was divided into a training set, used for parameter setting, and a test set, used to evaluate the performance of the proposed approach. We also used the DiaretDB1 public database to evaluate the generalization ability of the proposed method. It should be noted that the images if DiaretDB1 were not used for model building. They were only employed to assess the performance of the final method on a new set of images obtained with different protocols, resolution and quality. Additionally, the evaluation on this public data set allowed us to directly compare our results with some previous studies.

Our private image data set consisted of 564 fundus images provided by the “Instituto de Oftalmobiología Aplicada” (IOBA) of the University of Valladolid (Valladolid, Spain) and the “Hospital Clínico Universitario de Valladolid” (Valladolid, Spain). All subjects gave their informed consent to participate in the study. Our research was conducted in accordance with the Declaration of Helsinki, and the protocol was approved by the Ethics Committee at “Hospital Clínico Universitario de Valladolid”. The digital images were captured using a Topcon TRC-NW400 automatic retinal camera (Topcon Medical Systems, Inc., Oakland, NJ, USA) at a 45 degree FOV. They had a resolution of 1956 × 1934 pixels with a 24-bit JPEG format. Images were captured using the two-field protocol adopted by National Service Framework for Diabetes in the United Kingdom for DR screening [48]. This protocol involves the acquisition of a fovea-centered image and an optic disc-centered image per eye [48]. Thus, four images were acquired for the majority of patients involved in the study. However, image capture-related problems prevented some images to be recorded for certain patients. Among the complete set of 564 images, 239 had some type of RLs. The remaining 325 images lacked DR lesions. The database was randomly divided into two balanced sets. The training set (281 images) allowed us to develop the method and optimize its parameters. The test set (283 images) was used to evaluate the performance of the method. All the RLs in our database were manually annotated in detail by an ophthalmologist.

The DiaretDB1 database is composed of 89 images captured in the Kuopio University Hospital [49]. It was divided into a training set (28 images) and a test set (61 images). In this study, the proposed method has been configured and trained using the 281 images in the training set of our private database. For this reason, the training images of DiaretDB1 were not employed. We only used the 61 images in the test set of DiaretDB1 in order to assess the performance and robustness of the proposed method on a new set of images. In DiaretDB1, only one image per eye (macula centred) was captured with a 50° FOV. Image resolution was 1500 × 1552 pixels. The image ground truth of the lesions was roughly annotated by four experts using circles, ellipses and polygons [49].

### 3.2. Performance Assessment

The optimal parameters of the proposed algorithm were determined using the training set of our private database, composed of 281 fundus images. Table 2 compiles the chosen values for all of those parameters. The number of hidden neurons and the regularization parameter value were experimentally adjusted. We varied the value of those free parameters using 10-fold-cross-validation. We chose the parameter values that maximized the average accuracy over the validation set ($ACC_{NN}$), keeping the balance between the average sensitivity ($SE_{NN}$) and the average specificity ($SP_{NN}$) over the validation set.

After the parameters of the algorithm were set, we evaluated the effectiveness of our approach using the test set of our private database (283 images) and the test set of the DiaretDB1 database (61 images). We obtained the results on both databases in terms of two different criteria [32]:

- Pixel-based criterion: we considered all the pixels belonging to a correctly detected superpixel to be correct. A superpixel was correctly detected when, at least, one of its pixels was identified as a RL. Based on this criterion, we calculated the positive predictive value ($PPV_{p}$) and the sensitivity ($SE_{p}$) [50].
- Image-based criterion: frequently, the detection of very small reddish regions in some healthy images correspond to noisy spots. Therefore, they can be regarded as clinically non-significant and the image may be regarded as belonging to an eye with no signs of DR. Following the same approach used in previous studies [32,50], we considered an image pathological when, at least, 30 pixels in the whole image were detected as RLs. This corresponds to a very small fraction of the pixels of the image. Otherwise, the image was considered as containing no RLs. It should be noted that the image resolution in our database is higher than in previous studies [32,50]. This way, the value of 30 pixels is a more restrictive criterion in our study, since it represents a smaller fraction of the pixels in the image. This means that an image was considered as belonging to a healthy retina only when a very small proportion of its pixels (0.000008%) was detected as red lesion (RL). For the image-based criterion, the average sensitivity ($SE_{i}$), specificity ($SP_{i}$) and accuracy ($ACC_{i}$) over the test set were calculated.

### 3.3. Training

#### 3.3.1. Feature Selection

All 39 features described in Section 2.3.1 were studied in terms of relevancy and redundancy applying the FCBF algorithm. A total of 54233 superpixels were extracted from the training set, of which only 1783 corresponded to RL. To balance the two classes considered during training, we also randomly selected another 1783 superpixels that did not correspond to RLs. Thus, a vector of 39 features was calculated for each of the 3566 considered superpixels. It is important to note that feature vectors where normalized (mean = 0, standard deviation = 1) to improve the classification results [43].

After applying FCBF, the 16 features indicated in Table 1 were selected. It should be noted that the reduced feature set contained features related to shape, pixel intensities or the distance to the retinal structures. Hence, the selected features take into account the different distinctive characteristics of RLs.

#### 3.3.2. MLP Configuration

**Figure 6:**
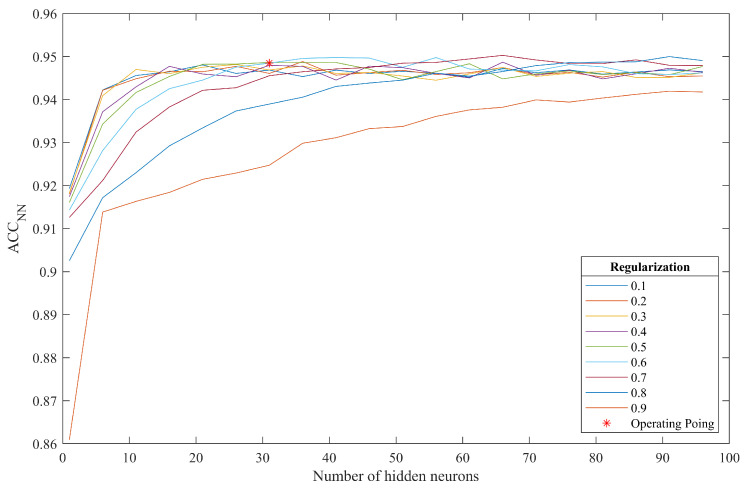
Accuracy curves obtained from MLP training.

We experimentally determined the number of neurons in the hidden layer and the value of the regularization parameter. The number of hidden neurons was analyzed in the range $n_{hid}=\left[1:1:100\right]$. The regularization parameter values were varied in the range υ=[0:0.1:1]. For each combination of $n_{hid}$ and υ, we obtained $ACC_{NN}$, $SE_{NN}$ and $SP_{NN}$. The values of $ACC_{NN}$ are shown in Figure 6. It can be seen that $ACC_{NN}$ stabilizes from $n_{hid}=30$ when υ<0.6. For larger values of υ, $ACC_{NN}$ decreases. Thus, we selected $n_{hid}=30$ and υ=0.6. At this operating point, we obtained $ACC_{NN}=94.50\%$, $SE_{NN}=93.24\%$ and $SP_{NN}=95.76\%$. Subsequently, we trained an MLP with $n_{hid}=30$ and υ=0.6 using the complete training set.

### 3.4. Results on the Test Set

**Figure 7:**
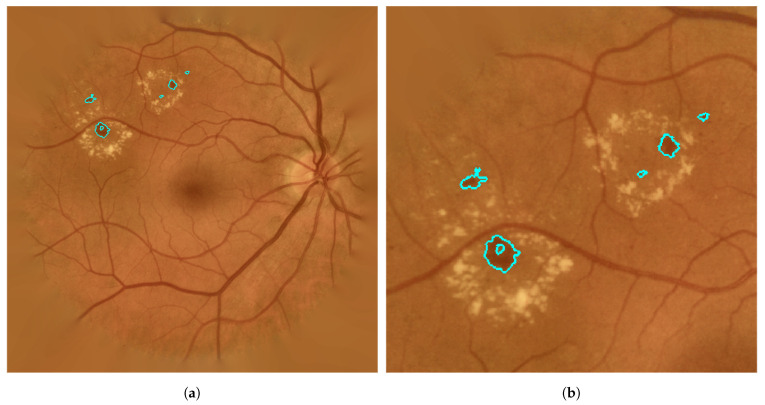
Definitively detected RLs after classification stage. (**a**) Example; (**b**) zoom in previous example.

After parameter setting during training, the method was applied on the images in the test set. The results of the automatic method were compared with the annotations of the ophthalmologist in terms of a lesion-based criterion and an image-based criterion [32]. The results are summarized in Table 3. The performance of the proposed method is illustrated in Figure 7, which corresponds to the example in Figure 4a.

### 3.5. Results on the DiaretDB1 Database

The proposed method was applied on the images in the test set of the DiaretDB1 database. The detected lesions from the automatic algorithm were compared with the image ground truth in terms of a lesion-based criterion and an image-based criterion [32]. The results are summarized in Table 3.

## 4. Discussion

In this study, we proposed an automatic method to detect RLs in fundus images based on superpixel classification. Firstly, a novel preprocessing was carried out to normalize the inter-image and intra-image appearance as well as to enhance the retinal structures. Secondly, the potential RL candidates were segmented. For this task, dark pixel detection was performed. Then, we separated the different elements of the image on the basis of their edges using the ERS method. RL candidates were later combined to reduce inaccurate fragmentation within structures. A set of 39 features related to shape, pixel intensity or structure distances was extracted from each superpixel. Then, a subset of 16 features was selected based on their relevancy and redundancy using the FCBF method. Finally, each candidate was classified by an MLP to yield the final segmentation of RLs.

The detection of RLs in fundus images has been addressed in previous studies. Different methods can be found in the literature, including mathematical morphology, region growing, wavelet, pixel classification and hybrid approaches [7]. More recent studies used flooding [23] and deep learning [24] techniques. Commonly, these algorithms start by segmenting the normal anatomical structures. Particularly, the blood vessels are generally extracted since they can be easily detected as RLs [2]. An important contribution of this study is that non-RL regions are eliminated during the classification stage, avoiding the need for an independent method for blood vessel segmentation. It should also be noted that the proposed method includes a novel preprocessing algorithm to achieve intra-image and inter-image normalization. It includes several operations for retinal image enhancement: bright border artifact removal, retinal fundus extension outside the FOV, intra-image and inter-image illumination and color equalization, denoising and contrast enhancement. To the best of our knowledge, this is the first time that the proposed operations have been used together for fundus images. In this sense, this preprocessing algorithm could be useful for different retinal image analysis problems beyond RL detection. Additionally, almost all of the previously proposed methods consider pixels as the basic unit in the image. However, superpixels are more consistent with human visual cognition. Unlike pixels, the superpixels provide a novel and meaningful representation of the natural entities of the image [25]. Superpixel segmentation for the detection of RLs in retinal images has been addressed in previous works since it can provide a significant representation of these lesions in the image [25,28]. In these studies, the SLIC method was chosen to segment image superpixels. However, this algorithm has important drawbacks, producing some under-segmented superpixels and poorer accuracy for border segmentation [29]. Therefore, we have followed a different approach to extract the superpixels in the image, using the ERS algorithm. To the best of our knowledge, this algorithm has never been applied in retinal image processing. We have verified that the segmentation accuracy improves when comparing the results of the ERS with the SLIC method [28] on the images in our database. Regarding computational cost, the ERS is a fast method, computing each image in less than 5 s using a CPU i7-7700@3.6 GHz. In order to improve the performance of the method, we have included a novel improvement to the ERS by combining the superpixels that belong to the same structure in order to reduce the number of candidates for the classification stage.

The proposed method was evaluated on a set of 283 fundus images, of which 120 presented RLs. Therefore, 163 images belonged to retinas lacking RLs. The images in our database showed a wide variability in terms of color, luminosity, contrast and varying levels of quality. Furthermore, different degrees of DR severity could be found and RLs were very variable in terms of appearance and size. The results obtained were measured with two different criteria. With the pixel-oriented criterion, a $SE_{p}$ of 81.43% and a $PPV_{p}$ of 86.59% were reached. With the image-oriented criterion, we obtained $SE_{i}=84.04\%$, $SP_{i}=85.00\%$ and $ACC_{i}=84.45\%$. These results are similar to those of other studies for RL detection according to the image-oriented criterion, as shown in Table 4. However, comparisons should be made with caution since the databases and the performance measures vary among studies. In the work of Seoud et al. [23] three different databases were used to evaluate their method in a per-image basis. Regarding Messidor database and the private CARA1006 database, they reached high $SE_{i}$ but an $SP_{i}$ of 50.00%. In the Erlangen database, the method obtained both a $SE_{i}$ and a $SP_{i}$ of 93.30%. However, this database was composed of only 45 images. Garcia et al. [32] tested their proposal in 65 fundus images from a private database. They obtained a $SE_{i}$ of 100% but an unbalanced $SP_{i}$ of 56.00%. In the study where SLIC superpixel segmentation was used, the proposed method reached $SE_{i}=83.30\%$ and $SP_{i}=97.30\%$ [25]. However, it was evaluated over only 49 images from DiaretDB1. Orlando et al. [24] used the Messidor data set to evaluate their method. Results showed a $SE_{i}=91.10\%$ when $SP_{i}=50.00\%$. In the work of Roychowdhuri et al. [51], the proposed method was evaluated using the DiaretDB1 database, obtaining $SE_{i}=75.50\%$ and $SP_{i}=93.73\%$. Sánchez et al. [52] validated their method using the Messidor data set. They reached $SP_{i}=92.20\%$ and $SP_{i}=50.00\%$. Niemeijer et al. [3] obtained $SP_{i}=100\%$ and $SP_{i}=93.73\%$ with pixel classification. However, they used a private database of only 100 images. The approach proposed by Grisan and Ruggeri showed $SP_{i}=99.00\%$ yet $SE_{i}=71.00\%$ [53]. Moreover, they used a private data set. Since our method has also been assessed on the test set of the public database DiaretDB1, a direct comparison with the methods proposed by Roychowdhuri et al. [51] and Zhou et al. [25] on the same database can be made. Roychowdhuri et al. [51] obtained $SE_{i}=75.50$%, lower than that obtained with our proposal ($SE_{i}=84.00\%$). However, they obtained $SP_{i}=93.73$%, which is higher than the results in our study ($SP_{i}=88.89$%). Similar results were obtained in the study by Zhou et al. [25]. Their $SE_{i}$ reached $SE_{i}=83.30$%, which is slightly lower than in our approach. In addition, their $SP_{i}$ also improves our results, reaching $SP_{i}=97.30$%. When comparing our results with previous approaches, it should be noted that the proposed method has been configured and trained using only images from our private database. These images differ from the images of the public database DiaretDB1 in multiple aspects. Firstly, the images in our database have a higher resolution. Secondly, they have been captured using a different protocol. Additionally, the FOV in the images of our private database is 45°, while the FOV in the images of DiaretDB1 is 50°. They also were selected with different quality criteria [49]. In spite of these differences, our results on the test set of DiaretDB1 were reasonably good, which confirms the robustness of the proposed method. It is also important to point out, for global comparisons, that our private database consisted of 564 fundus images with a resolution of 1956 × 1934 pixels. The performance criteria are also heterogeneous among studies. This is especially relevant when a region-based or pixel-based criterion is used. When comparing to the ophthalmologist annotations, the detected regions may overlap completely, partially or not at all with any RL. Thus, there is ambiguity between studies in considering whether a RL is correctly detected. In this sense, individual pixels belonging to an overlapping region could be sometimes considered as RLs or not depending on the criteria of the study.

**Figure 8:**
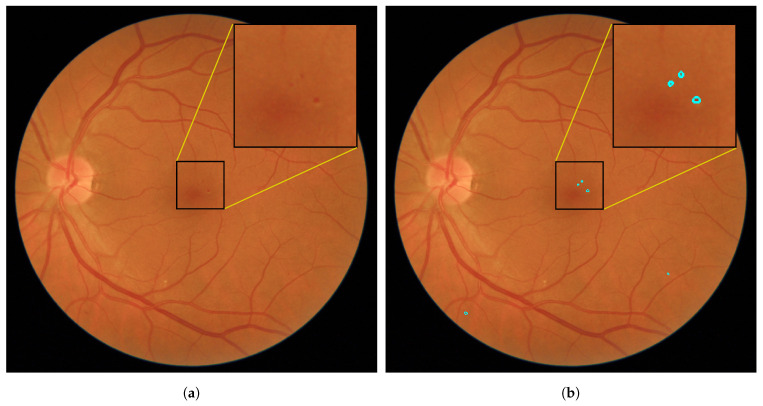
Fundus image example corresponding to a retina with MAs. (**a**) Original image; (**b**) detected RLs over the original image.

**Figure 9:**
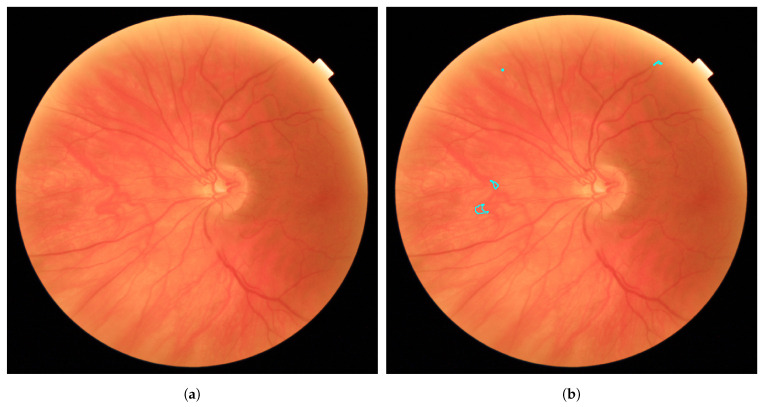
Fundus image example corresponding to a healthy retina. (**a**) Original image; (**b**) wrongly detected RLs over the original image.

The visual inspection of the results on the test images showed that the proposed approach adjusted considerably well to the edges of the true RLs (see Figure 7). Moreover, it is capable of detecting the smallest RLs. In Figure 8a, a fundus image with some MAs can be seen. Figure 8b shows how the proposed method correctly detects the MAs even when they are not sharp. However, in certain images, some regions were wrongly detected as RLs. In Figure 9a, we can see a fundus image corresponding to a healthy retina according to the expert annotations. Figure 9b shows the regions that the proposed method detects as RLs. In this image, the background shows a number of strongly visible choroidal vessels as well as a non-uniform texture. As a consequence, many superpixels were considered as RL candidates and our method fails to eliminate all of them as non-RLs.

The proposed method has other limitations that should be mentioned. In the first place, the ERS algorithm does not always properly manage to isolate the MAs or RLs of small size. The size of the superpixels is a commitment parameter. Larger values of *K* would make superpixels to better approximate small RLs. However, superpixels also become closer to the concept of individual pixels, losing the representation of the entities of the image [26,28]. In future works, we will try to detect MAs independently from HEs, adjusting the method parameters for each type of lesion. Solving the problem as two independent segmentation challenges allows us to adjust the parameters of the method more properly. Secondly, despite the combination of neighboring superpixels of similar color, many structures (some vessels, for instance) are divided into different superpixels. Ideally, a single superpixel should encompass the entire structure. However, this limitation also makes it easier to differentiate RLs that are very close to the vasculature. We will also consider extending the idea of superpixel classification to the segmentation of other lesions or retinal structures. Finally, although our database is larger than the databases employed in other studies [25,32], it would be desirable to increase the size of the database in future studies.

## 5. Conclusions

In summary, the proposed method deals with superpixels instead of pixels to identify the entities of the image. To group a set of pixels into a superpixel, we used the ERS method, which follows a clustering formulation based on an entropy measure. The concept of the superpixel is suitable to separate the different structures of the image. Likewise, the ERS algorithm is a fast and effective RL segmentation technique. In addition, dispensing of a prior vessel segmentation stage avoids the possible errors derived from this phase. Our results suggest that the proposed method could be useful for the detection of RLs in retinal images. Therefore, it could be a diagnostic aid for the early detection of DR, reducing the workload of specialists and improving the care of diabetic patients.

## Figures and Tables

**Table 1:** Extracted features. The last column indicate the selected features.

Feature Number	Description	Selected
1	Area of the region.	1
2	Width of the bounding box (smallest rectangle containing the region).	-
3	Heigh of the bounding box.	-
4	Area of the smallest convex hull (smallest convex polygon that can contain the region).	-
5	Eccentricity of the ellipse that has the same second-moments as the region.	5
6	Number of holes in the region.	6
7	Ratio of pixels in the region to pixels in the total bounding box.	7
8	Length of the major axis of the ellipse that with same normalized second central moments as the region.	8
9	Length of the minor axis of the ellipse that with same normalized second central moments as the region.	-
10	Distance around the boundary of the region (perimeter length).	-
11	Proportion of the pixels in the convex hull that are also in the region (solidity).	11
12–14	Mean of the pixels inside the region computed in the RGB channels of the image $I_{prep}$.	12,13
15–17	Median of the pixels inside the region computed in the RGB channels of the image $I_{prep}$.	-
18–20	Standard deviation of the pixels inside the region computed in the RGB channels of the image $I_{prep}$.	19
21–23	Entropy of the pixels inside the region computed in the RGB channels of the image $I_{prep}$.	21
24–26	Mean of the pixels inside the region computed in the RGB channels of the image $I_{dark}$.	-
27–29	Median of the pixels inside the region computed in the RGB channels of the image $I_{dark}$.	27,28
30–32	Standard deviation of the pixels inside the region computed in the RGB channels of the image $I_{dark}$.	-
33–35	Entropy of the pixels inside the region computed in the RGB channels of the image $I_{dark}$.	-
36	Mean of the pixels calculated in the border of the region applying Prewitt operator in the image $I_{prep}$ [40].	36
37	Mean of the pixels inside the region calculated in the result of applying multiscale line operator filters [41].	37
38	Distance to the center of the optic disc, calculated using [42].	38
39	Distance to the center of the fovea, calculated using [42].	39

**Table 2:** Values of the parameters of the proposed method.

Parameter	Value	Section
Denosing filter size	3 pixels	Preprocessing-denoising
*s*	$\left\{\frac{D}{48},\frac{D}{24},\frac{D}{12},\frac{D}{6},\frac{D}{3}\right\}$	Dark pixel detection
$\alpha_{s}$	$1-(3.84\frac{s}{D})$	Dark pixel detection
*K*	2000	Entropy Rate Superpixel Segmentation
λ	0.08	Entropy Rate Superpixel Segmentation
σ	2	Entropy Rate Superpixel Segmentation
Threshold	0.3	Candidate reduction
Color distance	0.24	Candidate reduction
$n_{hid}$	30	MLP Configuration
υ	0.6	MLP Configuration

**Table 3:** Results on the test set.

Database	Pixel-Based Criterion		Image-Based Criterion		
	${\mathrm{SE}}_{p}$	${\mathrm{PPV}}_{p}$	${\mathrm{SE}}_{i}$	${\mathrm{SP}}_{i}$	${\mathrm{ACC}}_{i}$
Private	81.43%	86.59%	84.04%	85.00%	84.45%
DiaretDB1	88.10%	93.10%	84.00%	88.89%	86.89%

**Table 4:** Performance comparison of some methods for the detection or RLs in fundus images according to the image-oriented criterion.

Authors	Method	Database	Nb. Images	${\mathrm{SE}}_{i}$	${\mathrm{SP}}_{i}$
Seoud et al. 2015 [23]	Flooding	Messidor	1200	93.90%	50.00%
Seoud et al. 2015 [23]	Flooding	Erlangen	45	93.30%	93.03%
Seoud et al. 2015 [23]	Flooding	CARA1006	1006	96.10%	50.00%
García et al. 2010 [32]	NNs	Private	115	100%	56.00%
Zhou et al. 2017 [25]	SLIC Superpixel	DiaretDB1	89	83.30%	97.30%
Orlando et al. 2018 [24]	Deep Learning	Messidor	1200	91.10%	50.00%
Roychowdhuri et al. 2012 [51]	Gaussian Mixture Models	DiaretDB1	89	75.50%	93.73%
Sánchez et al. 2011 [52]	Gaussian filter bank	Messidor	1200	92.20%	50.00%
Niemeijer et al. 2005 [3]	Pixel classification	Private	100	100%	87.00%
Grisan and Ruggeri 2005 [53]	Bayesian classification	Private	260	71.00%	99.00%
**Proposed method**	**Entropy Rate Superpixel**	**Private**	**564**	**84.04%**	**85.00%**
**Proposed method**	**Entropy Rate Superpixel**	**DiaretDB1**	**89**	**84.00%**	**88.89%**
